# Detection of genes positively selected in Cuban *Anolis* lizards that naturally inhabit hot and open areas and currently thrive in urban areas

**DOI:** 10.1002/ece3.7161

**Published:** 2021-01-29

**Authors:** Shunsuke Kanamori, Antonio Cádiz, Luis M. Díaz, Yuu Ishii, Takuro Nakayama, Masakado Kawata

**Affiliations:** ^1^ Graduate School of Life Sciences Tohoku University Sendai Japan; ^2^ Department of Biology University of Miami Coral Gables USA; ^3^ National Museum of Natural History of Cuba Havana Cuba

**Keywords:** *Anolis*, positive selection, thermal adaptation, transcriptome, urban tolerance

## Abstract

Species of *Anolis* lizards of the West Indies that naturally inhabit hot and open areas also tend to thrive in urban areas. In this study, transcriptome was sequenced for nine species of Cuban *Anolis* lizards that are closely related to each other, but inhabit different thermal microhabitats. Using PAML and HyPhy software, we attempted to identify genes and amino acid sites under positive selection in the common ancestral branch of *A. porcatus* and *A. allisoni*, and the branch of *A. sagrei*, which inhabit hot and open areas, and thrive in urban areas. Although there were no genes where positive selection was commonly detected on both of the tested branches, positive selection was detected in genes involved in the stress response (e.g., DNA damage and oxidative stress) and cardiac function, which could be related to adaptive evolution of tolerance to heat or ultraviolet radiation, on both branches. These findings suggest that adaptive evolution of the response to stress caused by heat or ultraviolet radiation might have occurred in ancestors of *Anolis* species inhabiting hot and open areas and might be related to the current thriving in urban areas of them.

## Graphical abstract

We constructed transcriptome sequences of nine species of Cuban Anolis lizards that are closely related to each other but live in different thermal microhabitats. Then, we attempted to detect genes and amino acid sites under positive selection for hot and open habitat dwelling species which thrive urban area.

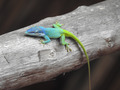

## 1 INTRODUCTION

Because forests have a thermal buffering capacity (De Frenne et al., 2019), a reduction in forest or canopy cover due to human activities can destroy the habitats of tree‐dwelling organisms, and would also result in severe temperature fluctuations on the ground. Urbanization, which is associated with the loss of forests, not only increases the amount of open areas with low thermal buffering capacity, but also changes the physical structure, humidity, light exposure, and predator–prey relationships (Forman, 2014). However, some species appear to rapidly respond to such changes and thrive in urban environments (e.g., Johnson & Munshi‐South, 2017; Winchell et al., 2016, 2018).

*Anolis* lizards inhabiting the Caribbean islands are model organisms for the study of mechanisms underlying adaptive radiation as different ecomorphs have adapted to distinct structural microhabitats (Losos, ). Some species of *Anolis* lizards thrive in the urban areas, while other species are rarely seen there (Winchell et al., 2020). Species of Caribbean *Anolis* lizards inhabiting hot and dry environments in the Lesser Antilles tend to have greater tolerance to urban areas (Winchell et al., 2020).

**FIGURE 1:**
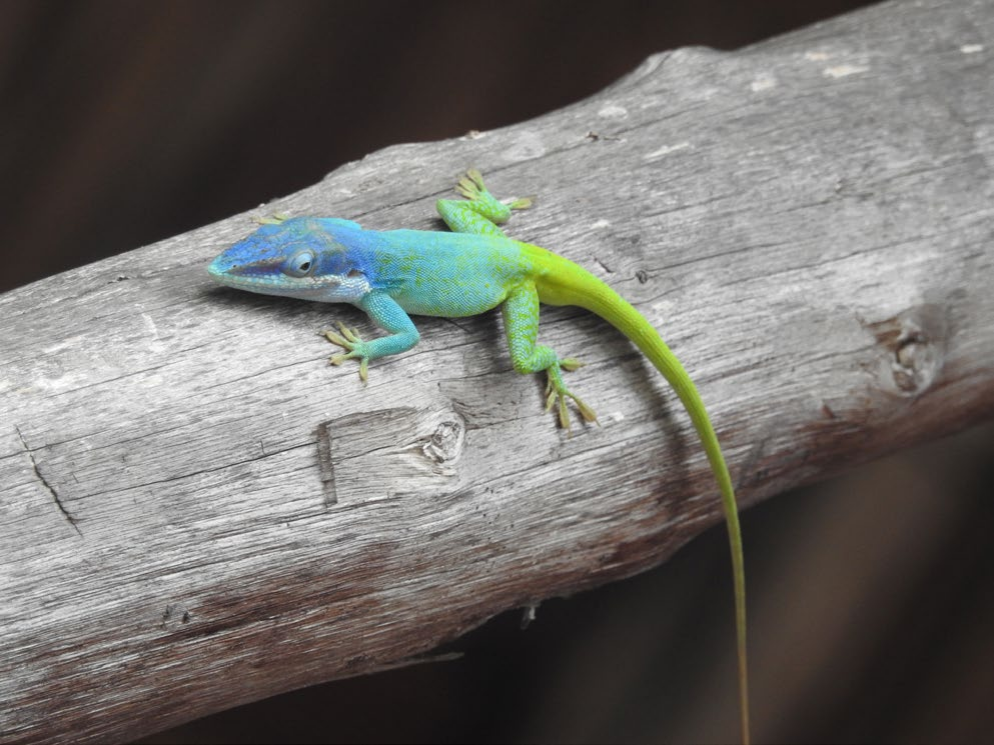
*Anolis allisoni* in the central Cuba. *A. allisoni* inhabits hot and open habitat, and often found in urban areas

Cuba has the largest number of endemic *Anolis* lizard species in the Caribbean (Cádiz et al., 2018; Losos, ; Uetz et al., ). In addition to structural habitat differentiation through evolution, ecomorphs segregate in different thermal microhabitats, such as shaded forests, forest margins, and open areas (Cádiz et al., 2013). Species of various lineages living in different thermal microhabitats, such as deep forests and open areas can be found in the same local area, which could be linked to local species diversity (Cádiz et al., 2013). For example, *A. allisoni* (Figure 1), *A. porcatus*, and *A. sagrei* are found in hot and open areas, while *A. alutaceus* and *A. isolepis*, *A. garridoi*, *A. allogus*, and *A. mestrei* are found in cool and shaded forests, and *A. homolechis* is found in intermediate forest margins (Rodríguez‐Schettino, 1999). *A. allogus*, *A. homolechis*, *A. mestrei*, and *A. sagrei* are all trunk‐ground species and can coexist sympatrically owing to differentiated thermal microhabitats (Cádiz et al., 2013; Losos, ; Ruibal, 1961; Rodríguez‐Schettino, 1999; Rodríguez‐Schettino et al., 2010). In Cuba, several species of *Anolis* lizards occupy urban areas. In the city of Havana, *A. porcatus* and *A. sagrei* are the most common in urban environments, including the downtown and residential areas, which are open areas with high solar radiation and temperatures, similar to their natural habitats. *A. allisoni* has been also introduced into such urban environments, although this species is not as common in Havana as *A. porcatus* and *A. sagrei*. In addition, *A. carolinensis*, a close relative of *A. porcatus* and the only *Anolis* lizard native to North America (Glor et al., 2005), has recently invaded the islands of Okinawa and Ogasawara (Japan), as well as the Hawaiian islands (USA) (Suzuki‐Ohno et al., 2017; Tamate et al., 2017), while *A. porcatus* and *A. allisoni* have been observed in the state of Florida (USA) (Donini & Allman, 2017; Kolbe et al., 2007). *A. sagrei* has also recently invaded various areas, including Florida, the Hawaiian islands, and the island of Taiwan (Kolbe et al., 2004). Traits related to the adaptation to hot and open habitats, and tolerance to urban environments may also be associated with invasiveness.

Several studies of the genetic basis underlying adaptation of Cuban *Anolis* lizards to different thermal microhabitats have been conducted by comparing *A. sagrei* inhabiting hot and open areas with *A. allogus* inhabiting cool and shaded forests and *A. homolechis* inhabiting forest margins. Akashi et al. (2018) analyzed the temperature at which these three species escape from heat source by behavioral experiment. Then, they expressed a putative molecular heat sensor, transient receptor potential ion channel ankyrin 1 (*trpa1*) of these three species ﻿on *Xenopus* oocytes and electrophysiologically quantified activation temperature of TRPA1 channel encoded by *trpa1* (Akashi et al., 2018). The results of their experiments showed that the temperature which elicits escaping behavior and thermal activation threshold of TRPA1 of *A. homolechis* and *A. sagrei* are higher than those of *A. allogus* (Akashi et al., 2018). In addition, Akashi et al. (2016) detected differentially expressed genes (DEGs) associated with adaptation to different thermal microhabitats and identified a gene associated with circadian regulation and metabolism, *nr1d1*, as a DEG with opposite expression patterns for the hot‐adapted *A. sagrei* and the cool‐adapted *A. allogus*. However, little progress has been made in the identification of genetic variants involved in the adaptation to different thermal microhabitats.

*Anolis cristatellus* recently expanded into urban areas on the island of Puerto Rico. Campbell‐Staton et al. (2020) investigated the adaptive evolution of this species to heat stress and found that nonsynonymous polymorphisms at one site within the protein synthesis gene *rars* are associated with plasticity of heat tolerance with parallel selection across multiple urban populations. *A. cristatellus* may originally have had the ability to adapt to a wide range of environments, from forests to urban areas. Winchell et al. (2020) suggested that species whose natural habitats are hot and dry tend to thrive in urban areas. Therefore, species that evolutionary adapt to open, hot, and dry habitats could have an innate ability to invade and colonize urban environments. Hence, to determine how certain species can thrive in urban environments, it is necessary to clarify the genetic changes that occurred during adaptive evolution to open and hot natural habitats.

**FIGURE 2:**
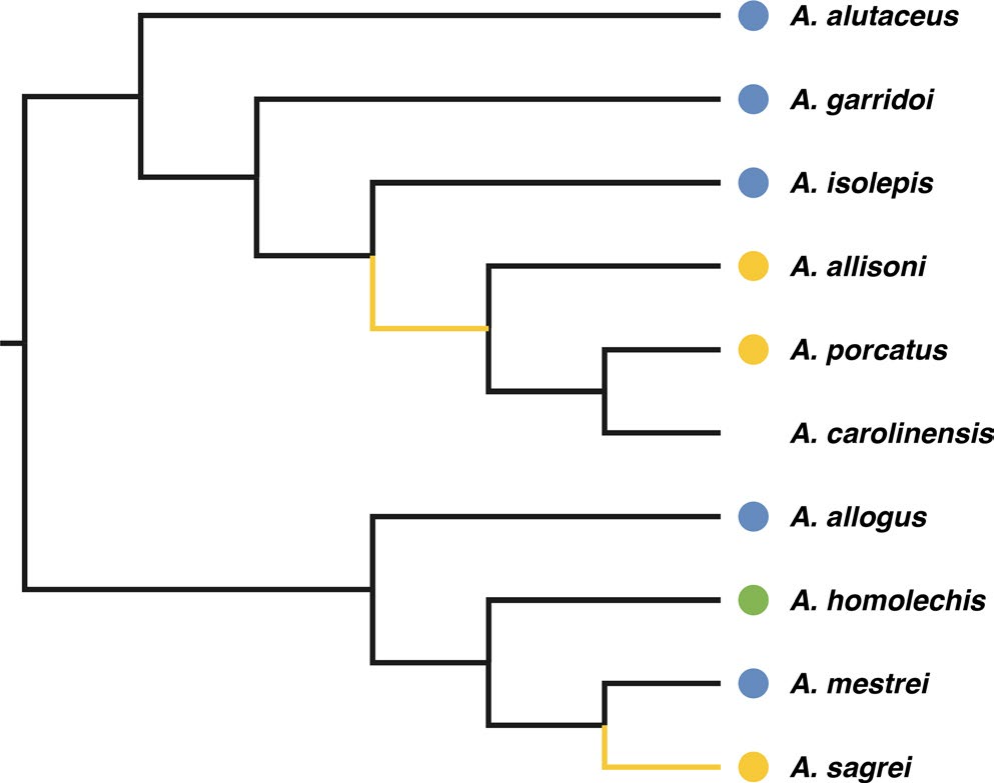
The phylogenetic relationships of nine Cuban *Anolis* lizards used to detect genes under positive selection and *A. carolinensis* used as reference species for ortholog detection and gene name assignment, and their natural habitats. The phylogenetic relationship of nine Cuban *Anolis* lizards is based on a reconstruction by Cádiz et al. (2018). The position of *A. carolinensis* is based on a reconstruction by Glor et al. (2005). Branches colored in yellow indicate foreground branches for detection of genes under positive selection by codeml and aBSREL. The color of the circle represents thermal microhabitat of each species, where blue indicates cool and shaded habitats, such as forests, green indicates intermediate forest margin habitats, and yellow indicates hot and open habitats

The purpose of this study was to detect genes that evolved under positive selection in Cuban *Anolis* lizards of different lineages that adapted to open and hot environments and are now thriving in urban areas. The estimated evolutionary history of urban tolerance liability associated with hot and dry conditions in natural habitats shows a possible evolutionary increase in urban tolerance in the common ancestor of *A. allisoni* and *A. porcatus*, and in *A. sagrei* (Winchell et al., 2020). In this study, we phylogenetically analyzed protein‐coding sequences in nine species of Cuban *Anolis* species: *A. alutaceus*, *A. garridoi*, *A. isolepis*, *A. allisoni*, *A. porcatus*, *A. allogus*, *A. homolechis*, *A. mestrei*, and *A. sagrei* and detected genes positively selected in the common ancestral branch of *A. allisoni* and *A. porcatus*, and in the *A. sagrei* branch (Figure 2).

## 2 MATERIALS AND METHODS

### 2.1 Sample preparation

In September 2016, individual specimens of *A. alutaceus*, *A. garridoi*, and *A. porcatus* were collected from Topes de Collantes nature reserve park (Escambray Mountains range), *A. isolepis* from Las Terrazas (Artemisa Province), *A. allisoni* from Trinidad (Sancti Spíritus Province), and *A. mestrei* from Soroa (Artemisa Province). All specimens were decapitated, immediately preserved in RNAlater® tissue storage reagent (Ambion, Foster City, CA, USA ), and brought to Japan in a cold condition. Of the six specimens, five were female, whereas the *A. allisoni* specimen was male. Sample collection in Cuba and export to Japan were approved by the Centro de Control y Gestión Ambiental of the Agencia de Medio Ambiente de Cuba.

### 2.2 RNA extraction, RNA sequencing (RNA‐seq), and de novo assembly

According to a previous report, genes expressed in the brain, liver, and skin of three *Anolis* lizards (*A. allogus*, *A. homolechis*, and *A. sagrei*) differ to some extent (Akashi et al., 2016). To sequence by RNA‐seq and comprehensively detect positive selection for a wide range of genes, it is necessary to extract RNA from multiple tissues that have a difference in the expressed genes. Therefore, we extracted RNA from those three tissues (i.e., brain, liver, and skin). The excised tissues were stored in RNAlater®. RNA was extracted from each tissue sample using an RNeasy Plus Mini Kit (Qiagen, Hilden, Germany) or a Maxwell RSC Plant RNA Kit with the Maxwell® RSC Instrument AS4500 (Promega, Madison, WI, USA) in accordance with the manufacturers’ protocols. RNA concentrations of the sample solutions were measured with an Agilent Bioanalyzer using an Agilent RNA 6000 Nano Kit (Agilent Technologies, Inc., Santa Clara, CA, USA), and RNA from each individual's tissues were mixed in equal amounts. Concentrations of the final RNA solutions ranged from 36 to 150 ng/µl. Library preparation and RNA‐seq were performed by the Beijing Genomics Institute (Shenzhen, China) or Novogen (Pledran, France) under mediation by Filgen, Inc. (Aichi, Japan). The constructed libraries were sequenced using a HiSeq 4000 or NovaSeq 6000 sequencing system (both, Illumina, Inc., San Diego, CA, USA). The reads from the libraries were assembled de novo using Trinity software version 2.8.5 (Grabherr et al., 2011). RNA‐seq reads of individuals of *A. allogus, A. homolechis*, and *A. sagrei* previously provided by Akashi et al. (2016) were downloaded from the Sequence Read Archive of the DNA Data Bank of Japan (DDBJ) (accession no. DRA004457) and similarly assembled. The isolates of *A. allogus*, *A. homolechis*, and *A. sagrei* individuals were named “a_allogus,” “a_homolechis,” and “a_sagrei,” respectively (Table 1A). The quality and completeness of the de novo assembled transcriptomes were evaluated using Transrate (Smith‐Unna et al., 2016) with a protein sequence set of *A. carolinensis* obtained from the Ensembl 99 as a reference and BUSCO (Simão et al., 2015) using the ortholog database for vertebrates, respectively.

**TABLE 1:** (A) Downloaded RNA‐seq data generated by Akashi et al. (2016) and (B) results of RNA‐seq performed in this study

(A) Downloaded RNA‐seq data generated by Akashi et al. (2016)				
Species	Millions of reads	Total length of reads (Mb)	Run IDs in the DDBJ Sequence Read Archive (Accession number: DRA004457)	The isolate name of individual
*A. allogus*	108	10,783	DRR055059, DRR055051, DRR055067	a_allogus
*A. homolechis*	76	7,611	DRR055075, DRR055083, DRR055091	a_homolechis
*A. sagrei*	99	9,945	DRR055099, DRR055107, DRR055115	a_sagrei

(B) Results of RNA‐seq performed in this study		
Species	Millions of reads	Total length of reads (Mb)
*A. alutaceus*	53	5,345
*A. garridoi*	55	5,525
*A. isolepis*	49	7,345
*A. allisoni*	53	7,891
*A. porcatus*	48	7,141
*A. mestrei*	54	5,366

### 2.3 Ortholog detection and multiple alignment

For the transcriptome of each species, coding sequences (CDSs) were predicted and translated into protein sequences using the TransDecoder program implemented in Trinity software (Grabherr et al., 2011). To estimate homologous protein pairs, homology searches were performed reciprocally between the predicted proteins and the *A. carolinensis* protein sequence set obtained from Ensembl 99 using National Center for Biotechnology Information (NCBI) Blast+ (Camacho et al., 2009) (e‐value < 10^–15^). If multiple protein sequences were registered for one gene of *A. carolinensis* in the Ensembl 99 database, the longest one was used. The protein sequences of each species that were reciprocally homologous with a protein sequence of *A. carolinensis* were retrieved and used as an ortholog group together with the *A. carolinensis* sequence in subsequent analyses. The annotation of the *A. carolinensis* ortholog in Ensemble 99 was adopted for gene name encoding these proteins. If a gene name was not annotated to an *A. carolinensis* ortholog in Ensembl 99, the gene name of a human or chicken ortholog for the gene in Ensembl 99 was used. For genes where positive selection was detected, if gene name was not annotated to the ortholog of *A. carolinensis*, human or chicken in Ensembl 99, we conducted homology search by Web BLAST (https://blast.ncbi.nlm.nih.gov/Blast.cgi). Then, if the protein sequence of the *A. carolinensis* ortholog registered in Ensembl 99 best hit a gene of *A. carolinensis* with gene name registered in NCBI, the gene name of the gene registered in NCBI was adopted. The orthologous CDSs from multiple species were aligned together with a codon model using the PRANK multiple alignment program without a user‐specified initial guide tree (Löytynoja & Goldman, 2005). Then, sites that were gaps in more than two species were removed, and sequences with lengths after removal of the gap sites that were less than half those of *A. carolinensis* were excluded from further analysis of positive selection. A maximum likelihood phylogenetic tree for each ortholog group was reconstructed using RAxML with GTR‐GAMMA model (Stamatakis, 2014).

### 2.4 Detection of positive selection

Positive selection was detected using two software packages that implemented the branch site model: codeml from the PAML package (Yang, 2007) and aBSREL (Smith et al., 2015) from HyPhy package (Pond & Muse, 2005). For codeml, two models were fit and tested based on the null and alternative hypotheses of sequence evolution. The null hypothesis assumed that the gene evolved neutrally and the value of ω (i.e., d*N*/d*S*) was = 1 in all branches (Model A1: model = 2, NSsites = 2, fix_omega = 1, omega = 1), while the alternative hypothesis assumed that the ω value was > 1 in the foreground branch (Model A: model = 2, NSsites = 2, fix_omega = 0, omega = 1). The *p*‐values were calculated using the chi‐squared test with the maximum log‐likelihood of these two models. aBSREL (Smith et al., 2015) implemented in the HyPhy package (Pond & Muse, 2005) was used to test the null and alternative hypotheses with the same foreground branches used for codeml analysis. For detection of positive selection using codeml, two separate analyses were performed for each gene with the common ancestral branch of *A. allisoni* and *A. porcatus* or the *A. sagrei* branch as the foreground branch. Analysis by aBSREL detects genes under positive selection with a branch site model but focuses on the variation in the proportion of sites classified by d*N*/d*S* values. The *p*‐value of the likelihood ratio test was calculated by the program. Because aBSREL can assume different proportions of sites classified by d*N*/d*S* values on all the branches, and even if multiple branches are simultaneously set as foreground branches, a positive selection is independently detected in each branch. Therefore, for the detection of positive selection on each gene using aBSREL, the common ancestral branch of *A. allisoni* and *A. porcatus* and the *A. sagrei* branch are simultaneously set as foreground branches. The gene tree was used as an input tree for both the codeml and aBSREL analyses because, as has been pointed out, use of a species tree as a reference tree can increase the false positive rate of positive selection when the gene tree is discordant with the species tree (Mendes & Hahn, 2016). The q‐values were calculated from a collection of *p*‐values for each analysis using codeml and aBSREL in accordance with the method described by Storey et al. (2020), and genes were considered as candidates where positive selection occurred in the foreground branches when the q‐value obtained from codeml or aBSREL analysis was less than 0.05. If an amino acid site was assigned a posterior probability > 95% by Bayes empirical Bayes (BEB) analysis (Yang et al., 2005) in codeml, which infers amino acid sites under positive selection, and the amino acid of this site of *A. allisoni* and *A. porcatus*, or *A. sagrei*, differed from that of other forest‐dwelling and forest margin‐dwelling species, the site was considered a candidate under positive selection (hereafter, referred to as “a selection site”). The enrichment of gene ontology in genes, where positive selection was detected, was examined using PANTHER (Thomas et al., 2003).

### 2.5 Prediction of the impact of amino acid substitutions at selection sites

The potential effect of amino acid substitutions at selection sites on the biological function of proteins encoded by genes, where positive selection was detected, was predicted using SIFT (Kumar et al., 2009) with the vertebrate subsections of UniProtKB and Provean (Choi et al., 2012) with NCBI nr protein database. Those two software tools calculate the impact of amino acid substitutions on the protein in the context of evolutionary conservation. For each gene, protein sequence of *A. isolepis* was used as query. When the SIFT score was less than 0.05 or the Provean score was less than −2.5, the amino acid substitution at the BEB selective site was considered to have a significant impact.

## 3 RESULTS

### 3.1 RNA‐seq, de novo transcriptome assembly, and ortholog detection

RNA‐seq produced reads with a total length of 5,345–7,891 Mb for *A. alutaceus*, *A. garridoi*, *A. isolepis*, *A. allisoni*, *A. porcatus*, and *A. mestrei* (Table 1B). De novo assembly with Trinity using the RNA‐seq reads yielded 59,655–292,951 contigs. The Transrate assembly score and the completeness of the vertebrate BUSCOs for the obtained de novo assembly of transcriptomes were 0.0617–0.1761 and 61.2%–93.8%, respectively (Table 2). 23,698–75,370 CDSs were predicted for these contigs of each species. From these CDSs, 9,076–13,254 reciprocal orthologs for *A. carolinensis* for each species were obtained (Table 2). Finally, 7,113 ortholog groups that were reciprocally homologous with a common protein sequence of *A. carolinensis* in all nine species were identified. As a result of alignment and removal of the gap sites, 6,801 ortholog groups had alignment lengths greater than half the sequence length of *A. carolinensis*. Gene trees were constructed for these 6,801 ortholog groups. As a result, a gene tree, in which *A. allisoni* and *A. porcatus* were monophyletic, was reconstructed for 5,962 ortholog groups, which were assessed for positive selection.

**TABLE 2:** Results of assessment of the quality (Transrate assembly score) and the completeness (BUSCO metrics) of de novo transcriptome assemblies, and CDS prediction and reciprocal ortholog detection with *A. carolinensis*

Species	Number of contigs	Transrate assembly score	BUSCO metrics					Number of CDSs	Number of reciprocal orthologs with *A. carolinensis*
			% Complete	% Single‐copy	% Duplicated	% Fragmented	% Missing		
*A. alutaceus*	74,872	0.129	76.0	53.6	22.4	14.0	10.0	32,930	11,423
*A. garridoi*	59,655	0.1157	61.2	45.6	15.6	17.5	21.3	23,698	9,076
*A. isolepis*	187,177	0.0912	91.7	51.8	39.9	5.4	2.9	55,725	13,232
*A. allisoni*	292,951	0.0742	93.4	45.2	48.2	3.6	3.0	75,370	13,254
*A. porcatus*	242,827	0.0617	91.8	43.7	48.1	4.8	3.4	64,092	12,941
*A. allogus*	195,312	0.0926	93.5	55.6	37.9	3.2	3.3	56,329	13,066
*A. homolechis*	153,458	0.1024	92.2	56.0	36.2	3.9	3.9	49,053	12,817
*A. mestrei*	68,165	0.1761	66.2	51.3	14.9	16.6	17.2	26,437	9,704
*A. sagrei*	193,035	0.0786	93.8	51.0	42.8	3.1	3.1	60,396	13,171

### 3.2 Genes and amino acid sites with positive selection detected in lineages that inhabit hot and open areas, and thrive in urban areas

Genes under positive selection were detected in the common ancestral branch of *A. allisoni* and *A. porcatus*, and the *A. sagrei* branch that naturally inhabit hot and open areas, and thrive in urban areas. In the common ancestral branch of *A. allisoni* and *A. porcatus*, positive selection was detected in seven genes by either codeml or aBSREL analysis (Table 3A, Table S1). aBSREL analysis alone detected a positive selection in two of these genes (*baz1b* and *atp2a1*), whereas both analyses detected a positive selection in five of these genes (*trpm7*, *mxi1*, *trim39*, *abcb6*, and *eif3a*) (Table 3A, Table S1). In the *A. sagrei* branch, positive selection was detected in 14 genes by either codeml or aBSREL analysis (Table 3B, Table S1). Of these, positive selection was detected by the codeml analysis alone in four genes (*hgh1*, *tent4a*, *gba*, and *agap2*), by aBSREL analysis alone in three genes (*utp3*, *tex9*, and *pnpla6*), and by both analyses in seven genes (*cadm1*, *terf2*, *adgrl4*, *sptbn2*, *mef2a*, *tlcd3a*, and *c10orf88*) (Table 3B, Table S1). There was no common gene where positive selection was detected in the common ancestral branch of *A. allisoni* and *A. porcatus*, and the *A. sagrei* branch. Of seven genes where positive selection was detected in the common ancestral branch of *A. allisoni* and *A. porcatus*, selection sites (defined in the methods) were detected in *mxi1*, *trim39*, *abcb6*, and *eif3a* (Table 4). In 14 genes where positive selection was detected in the *A. sagrei* branch, no selection sites were detected. No significant enrichment of gene ontology with a *p*‐value of < .05 after correction of the false discovery rate was detected when the enrichment of gene ontology of genes where positive selection was detected in the two foreground branches was examined.

**TABLE 3:** The results of codeml and aBSREL analyses for genes, where positive selection was detected in (A) the common ancestral branch of *A. allisoni* and *A. porcatus*, and (B) the *A. sagrei* branch

(A) In the common ancestral branch of *A. allisoni* and *A. porcatus*									
Ensembl gene ID	Gene name	codeml in PAML					aBSREL in HyPhy		
		d*N*/d*S* of site class 2a and 2b	Log‐likelihood of alternative model	Log‐likelihood of null model	*p*‐value	*q*‐value	Max. d*N*/d*S*	*p*‐value	*q*‐value
ENSACAG00000003894	*baz1b* *	276.09	−9662.264856	−9667.036107	.0020	0.69	93.84	3.9E−05	0.034
ENSACAG00000003845	*trpm7*	999	−7342.358358	−7351.598684	1.7E−05	0.026	>1,000	2.0E−05	0.024
ENSACAG00000005552	*atp2a1*	1	−5253.5323	−5253.5323	1	1	>1,000	4.0E−05	0.034
ENSACAG00000006009	*mxi1*	479.33	−1440.046777	−1453.803647	1.6E−07	4.7E−04	718.58	1.6E−08	4.6E−05
ENSACAG00000011364	*trim39* *	23.51	−4298.903659	−4309.082467	6.4E−06	0.013	19.8	5.2E−08	1.0E−04
ENSACAG00000013017	*abcb6*	71.00	−6127.686384	−6136.507074	2.7E−05	0.032	>1,000	3.7E−06	0.0054
ENSACAG00000016784	*eif3a*	999	−8517.832307	−8562.51281	3.3E−21	2.0E−17	>1,000	0	0

(B) In the *A. sagrei* branch									
Ensembl gene ID	Gene name	codeml in PAML					aBSREL in HyPhy		
		d*N*/d*S* of site class 2a and 2b	Log‐likelihood of alternative model	Log‐likelihood of null model	*p*‐value	*q*‐value	Max. d*N*/d*S*	*p*‐value	*q*‐value
ENSACAG00000000541	*hgh1*	248.97	−2962.820331	−2973.425084	4.1E−06	0.0049	77.83	9.9E−04	0.23
ENSACAG00000000497	*tent4a*	157.45	−3446.357754	−3455.33254	2.3E−05	0.015	75.24	3.5E−04	0.10
ENSACAG00000001070	*cadm1*	999	−3015.023341	−3026.063567	2.6E−06	0.0039	713.29	1.4E−07	2.1E−04
ENSACAG00000003497	*terf2*	51.35	−3873.171509	−3896.181653	1.2E−11	2.3E−08	25.82	1.2E−09	2.3E−06
ENSACAG00000006559	*adgrl4*	999	−3497.926451	−3506.395159	3.9E−05	0.023	529.36	4.2E−05	0.028
ENSACAG00000007494	*sptbn2*	186.21	−15288.5029	−15407.79953	8.0E−54	4.8E−50	>1,000	0	0
ENSACAG00000009923	*mef2a*	525.39	−2803.399719	−2813.577961	6.4E−06	0.0064	469.4	9.2E−06	0.0092
ENSACAG00000010101	*gba* †	999	−5738.525135	−5748.042419	1.3E−05	0.0096	1	1	1
ENSACAG00000012860	*utp3*	89.36	−3157.86861	−3162.438382	.0025	0.44	134.39	8.2E−05	0.049
ENSACAG00000013313	*agap2*	1	−8855.973946	−8881.315423	1.1E−12	3.2E−09	>1,000	0.45	1
ENSACAG00000014134	*tex9*	1	−2855.807071	−2855.807071	1	1	173.74	1.2E−05	0.0098
ENSACAG00000016007	*tlcd3a*	999	−1687.940639	−1696.267181	4.5E−05	0.024	113.01	2.2E−05	0.017
ENSACAG00000016134	*pnpla6*	1	−10725.9462	−10725.9462	1	1	>1,000	5.7E−12	1.7E−08
ENSACAG00000017448	*c10orf88*	645.25	−3017.584938	−3027.286087	1.0E−05	0.0090	>1,000	7.0E−06	0.0084

* Indicates gene names not annotated to the ortholog of *A. carolinensis* but assigned using the results of homology searches using Web BLAST. Gene names that were not annotated to the ortholog of *A. carolinensis* but were annotated to human orthologs are indicated by †.

**TABLE 4:** Posterior probability of BEB analysis, and SIFT and Provean score of selection sites for genes, where positive selection was detected in the common ancestral branch of *A. allisoni* and *A. porcatus*

Gene name	Amino acid position in protein sequence of *A. isolepis*	Amino acid			Posterior probability of BEB analysis (%)	SIFT score	Provean score
		*A. isolepis*	*A. allisoni*	*A. porcatus*			
*mxi1*	83	V	I	I	95.8	0.08	−0.8
	85	I	R	R	98.6	0.4	−1
	91	S	E	E	99.8	0.48	−0.3
	95	Q	R	R	96.6	0.82	−0.4
	96	I	R	R	97.3	0.61	−0.333
*trim39* *	274	M	C	C	98	0.18	−1.667
*abcb6*	538	W	F	F	96.8	0.01	−9
*eif3a*	1,108	G	P	P	98.2	0.07	−0.3
	1,109	P	S	S	97	0.11	3.067
	1,112	G	N	N	96.5	0.79	1.6
	1,148	P	G	G	98.2	1	0.933
	1,152	*N*	G	G	96.8	0.85	−4.667
	1,153	T	L	L	99.2	0.41	−1.233

* Indicates gene names not annotated to the ortholog of *A. carolinensis* but assigned using the results of homology searches using Web BLAST.

In *mxi1*, *trim39*, *abcb6*, and *eif3a* where positive selection was detected in the common ancestral branch of *A. allisoni* and *A. porcatus*, the impact of amino acid substitutions of selection sites was predicted. As a result, of those selection sites, a significant impact was detected by both SIFT and Provean analysis at one selection site in *abcb6*, and a significant impact was detected only by Provean analysis at one selection site in *eif3a* (Table 4).

## 4 DISCUSSION

In this study, we detected genes under positive selection at the ancestral branches of Cuban *Anolis* lizards that dwell in hot and open areas and thrive in urban areas. We assumed that adaptive evolution to hot and open areas in the past allowed the tolerance of current hot and open urban environments because the species of Caribbean *Anolis* lizards that inhabit hot and dry environments and maintain high body temperatures, tend to have a greater tolerance to urban areas (Winchell et al., 2020). It is unclear whether adaptive evolution to hot and open habitats occurred within the two branches that we set as foreground branches, and it is possible that adaptive evolution to hot and open areas had occurred at more ancestral points. Because it is difficult to collect data and accurately classify thermal microhabitats for a large number of species in the genus *Anolis*, including species out of Cuba, it was not possible to identify the branches of species that underwent evolutionary changes to adapt to thermal microhabitats. However, Winchell et al. (2020) indicated that the liability of urban tolerance evolved in the common ancestor of *A. allisoni* and *A. porcatus*, and an ancestor of *A. sagrei*, and noted that urban tolerance is associated with hot and dry conditions of natural habitats. Because these lizards have only encountered human‐generated environments very recently, this relatively short period of time may have been insufficient for lizards to adapt to urban environments. Therefore, we detected genes positively selected at the focal branches using codeml and aBSREL to elucidate the genetic basis of adaptive evolution to hot and open areas, which allows tolerance of current urban environments.

There was no gene where positive selection was commonly detected with a false discovery rate of <0.05 in both the common ancestral branch of *A. allisoni* and *A. porcatus*, and that of *A. sagrei*, which inhabit hot and open areas, and thrive in urban areas. However, positive selection was detected in genes involved in the stress response (e.g., DNA damage and oxidative stress) and cardiac function in both branches. Species inhabiting hot and open areas could be exposed to heat stress and ultraviolet radiation, which can cause DNA damage and oxidative stress (Belhadj Slimen et al., 2014; Cadet & Wagner, 2013). Thus, genes related to the responses to DNA damage and oxidative stress might contribute to adaptive evolution to heat and ultraviolet radiation. *baz1b* and *trim39*, where positive selection was detected in the common ancestral branch of *A. allisoni* and *A. porcatus*, and *terf2*, in the *A. sagrei* branch appear to be involved in DNA damage repair. BAZ1B encoded by *baz1b* promotes recovery after DNA damage (Oppikofer et al., 2017), TRIM39 encoded by *trim39* regulates the balance between cytostasis and apoptosis after DNA damage (Zhang et al., 2012), and TERF2 encoded by *terf2* is involved in recognition of genomic double‐strand breaks as an early response to DNA damage (Bradshaw et al., 2005). *trpm7*, *mxi1* and *abcb6*, detected in the common ancestral branch of *A. allisoni* and *A. porcatus*, and *gba* and *c10orf88* in the *A. sagrei* branch might be related to response to oxidative stress. TRPM7 encoded by *trpm7* is activated by oxidative stress (Abiria et al., 2017) and mediates oxidative stress‐induced anoxic neuronal death (Aarts et al., 2003). MXI1 encoded by *mxi1* is thought to be involved in the protection of cells from apoptosis (Corn et al., 2005) and prevention of excessive production of reactive oxygen species (Delpuech et al., 2007). ABCB6 encoded by *abcb6* is thought to protect cells from peroxide‐induced stress (Lynch et al., 2009). PAAT encoded by *c10orf88* plays an important role in the maintenance of mitochondrial homeostasis, while the depletion of PAAT promotes oxidative stress‐induced cell death and mitochondrial DNA damage (Yang et al., 2014). *gba* might also be related to oxidative stress (Cleeter et al., 2013), but it may contribute to epidermal tolerance to desiccation since the expression of this gene is increased by epidermal water loss (Buraczewska et al., 2009). In addition, *eif3a*, in the common ancestral branch of *A. allisoni* and *A. porcatus*, and *mef2a*, in the *A. sagrei* branch, are involved in the response to cellular stress, such as iron depletion and hypoxia (Lane et al., 2013; Zhao et al., 1999).

The physiological limitation in thermal tolerance is thought to be related to cardiac function (Casselman et al., 2012; Pörtner et al., 2017). The expression of sarcoplasmic/endoplasmic reticulum calcium ATPase encoded by *atp2a1*, detected in the common ancestral branch of *A. allisoni* and *A. porcatus*, plays an important role in thermal acclimation of cardiac function (Korajoki & Vornanen, 2012, 2013) and a deficiency of ELTD1 encoded by *adgrl4*, in the *A. sagrei* branch, has been reported to exacerbate cardiac function (Xiao et al., 2012).

Of the 21 genes where positive selection was detected in the common ancestral branch of *A. allisoni* and *A. porcatus* or the *A. sagrei* branch, selection sites were detected in *mxi1*, *trim39*, *abcb6*, and *eif3a*, and a significant impact was detected at one of the selection sites of *abcb6* and *eif3a*. An amino acid variation at these sites would be a priority target to experimentally search for mutations related to adaptive evolution to hot and open habitats, and urban tolerance. However, future studies are necessary to examine how these amino acid mutations affect the living body.

Positive selection was not commonly detected on both of the two tested lineages in any of 5,962 genes examined in total. This result suggests that there might not be many common genes that were subject to positive selection in those two lineages. Corbett‐Detig et al. (2020) recently suggested that the convergent evolution of morphology and behavior in *Anolis* lizards may involve the evolution of many different protein or amino acid sites. Thus, adaptive evolution to hot and open habitats in different species might have been caused by the evolution of many different proteins or amino acid sites. However, many genes were excluded from analyses in this study (e.g., because a gene sequence for one or more species was not obtained or multiple alignment for a gene was significantly shortened in lengths after the gap removal). Completion of gene sequencing of these species is therefore needed for more comprehensive analysis.

## 5 CONCLUSION

In this study, positive selection was detected in seven and 14 genes in the common ancestral branch of *A. allisoni* and *A. porcatus*, and that of *A. sagrei* inhabiting hot and open area and thrive in urban areas, respectively. Although there were no genes where positive selection was commonly detected in both of the tested branches, positive selection was detected in genes involved in the stress response (e.g., DNA damage and oxidative stress) and cardiac function, which could be related to tolerance to high temperatures or UV radiation, on both branches. These results suggest that the positively selected changes of these genes might be associated with adaptation to high temperatures and open habitats, which in turn allows tolerance to hot and open urban habitats. The finding that positive selection of any of the 5,962 genes examined was not detected commonly in both of the tested lineages suggests that adaptive evolution of a common gene is rare. However, the focus of this study was limited to the coding regions and many genes were not able to be included in our analyses. Hence, further studies of more complete gene sets of many species, which can be obtained through genome sequencing, are required.

## Conflict of interest

None declared.

## AUTHOR CONTRIBUTION

**Shunsuke Kanamori:** Conceptualization (equal); Formal analysis (lead); Investigation (equal); Writing‐original draft (lead); Writing‐review & editing (equal). **Antonio Cadiz:** Investigation (equal); Writing‐review & editing (supporting). **Luis M Diaz:** Investigation (equal); Writing‐review & editing (supporting). **Yuu Ishii:** Formal analysis (supporting); Investigation (supporting); Writing‐review & editing (supporting). **Takuro Nakayama:** Formal analysis (supporting); Writing‐review & editing (supporting). **Masakado Kawata:** Funding acquisition (lead); Investigation (lead); Project administration (lead); Supervision (lead); Writing‐original draft (supporting); Writing‐review & editing (lead).

## Supporting information

Table S1

## Data Availability

RNA‐seq raw sequence reads are available through the DDBJ Sequence Read Archive under accession no. DRA010304. All the other data are included in Supplementary files.
